# Susceptibility of Field Populations of *Frankliniella intonsa* to Spinetoram, Imidacloprid, and Acetamiprid in Xinjiang Cotton Fields, China

**DOI:** 10.3390/insects16121234

**Published:** 2025-12-06

**Authors:** Xiaowei Li, Liqiu Wang, Wei Wang, Ju Yao, Farman Ullah, Chunmeng Li, Renfu Zhang, Yaobin Lu

**Affiliations:** 1 State Key Laboratory for Quality and Safety of Agro-Products, Key Laboratory of Biotechnology in Plant Protection of MOA of China and Zhejiang Province, Institute of Plant Protection and Microbiology, Zhejiang Academy of Agricultural Sciences, Hangzhou 310021, China; lixiaowei1005@163.com (X.L.); wanglq0909@163.com (L.W.); farmanullah787@gmail.com (F.U.); 2 Key Laboratory of Integrated Pest Management on Crop in Northwestern Oasis, Ministry of Agriculture and Rural Affairs, Institute of Plant Protection, Xinjiang Uygur Autonomous Region Academy of Agricultural Sciences, Urumqi 830092, China; wlzforever2004@sina.com (W.W.); yaoju500@sohu.com (J.Y.); 3 Jinyun County Wuyangwan Fruit and Vegetable Professional Cooperative, Lishui 321404, China; 4 Xianghu Laboratory, Institute of Bio-Interaction, Hangzhou 311231, China

**Keywords:** flower thrips, geographical variations, insecticide resistance

## Abstract

Thrips pests have become an increasing concern in cotton fields in Xinjiang. *Frankliniella intonsa* is the primary thrips species during the flowering and boll stages, causing boll stiffening or cracking. However, limited studies have been conducted on controlling *F. intonsa*. In this study, using a leaf-tube residue method, we evaluated the susceptibility of *F. intonsa* field populations to three insecticides (spinetoram, imidacloprid, and acetamiprid) collected from different geographical sites across major cotton planting areas in Xinjiang. The results showed that *F. intonsa* populations exhibited very high susceptibility to spinetoram, ranging from 0.003 mg L^−1^ in the Shihezi population to 0.036 mg L^−1^ in the Korla population. The susceptibility of *F. intonsa* to imidacloprid and acetamiprid varied considerably among different field populations, with the relative resistance of 33.00 and 25.71, respectively. Reduced susceptibility to all three insecticides was detected in the Korla and Manasi populations, highlighting the importance of implementing effective resistance management and alternative control strategies. These findings provide valuable information for the appropriate control of *F. intonsa* in Xinjiang.

## Simple Summary

Xinjiang is the largest cotton-producing area in China. In recent years, thrips pests have become an increasing concern in cotton fields in Xinjiang. Currently, controlling thrips in cotton fields relies heavily on the use of synthetic insecticides. However, limited studies have been reported on controlling thrips (*Frankliniella intonsa*) during flowering and boll stages. In this study, we evaluated the susceptibility of *F. intonsa* populations collected from different geographical sites across major cotton planting areas in Xinjiang to three insecticides: spinetoram, imidacloprid, and acetamiprid. We found that *F. intonsa* populations were highly susceptible to spinetoram, indicating that this insecticide could effectively control this pest in Xinjiang’s cotton fields. Susceptibility to imidacloprid and acetamiprid varied considerably among different field populations, with reduced susceptibility observed in locations such as Korla and Manasi. These results provide useful information for appropriate chemical control of *F. intonsa*.

## 1. Introduction

Xinjiang is the largest cotton-producing area in China, with its planting area and cotton yield accounting for 85.0% and 91.0% of the total nationwide in 2023, respectively [1]. Cotton has been one of the most important crops in Xinjiang, with its planting area (2.37 million hectares) accounting for 34.6% of the total crop planting area in the region, which far exceeds that of corn (1.44 million hectares) and wheat (1.21 million hectares) [1]. The layout of cotton planting areas in Xinjiang presents a clear trend of centralization, but in some areas, cotton fields are interwoven with other crops, such as corn, orchards, and vegetables. Cotton pests have been constant threats to cotton production in China, especially in Xinjiang [2,3]. In recent years, thrips pests have become a growing concern in Xinjiang’s cotton fields, with *Thrips tabaci* Lindeman and *Frankliniella intonsa* Trybom as the dominant species [4,5]. Thrips damage cotton plants at various growth stages. During the seedling stage, thrips (mainly *T. tabaci*) damage cotton by directly feeding on cotyledons and developing true leaves, resulting in malformation, tearing of leaves, and apical meristem damage. This leads to symptoms such as “headless cotton”, “multi-headed cotton”, and even seedling death [6,7]. During the flowering and boll stages, *F. intonsa* becomes the main thrips species. *Frankliniella intonsa* is a common flower-inhabiting thrips on many plants. It damages plants through direct feeding, oviposition, and indirect transmission of plant viruses. Its high reproductive capacity and short generation times lead to rapid population increase and severe damage to cotton flowers and bolls, causing boll stiffening or cracking, which directly affects the cotton yield [7,8].

Management of thrips pests in the field mainly depends on the frequent application of insecticides, including neonicotinoids, organophosphates, pyrethroids and carbamates [9,10], which has led to the development of insecticide resistance in many thrips species, such as *Frankliniella occidentalis* [11], *T. tabaci* [12,13], and *Thrips palmi* [14,15]. Understanding the susceptibility of thrips species in the field will assist in making informed decisions about chemical control. Currently, the control of thrips in cotton fields heavily relies on the application of synthetic insecticides [16]. Neonicotinoids, spinosyns, and diamides are commonly used insecticides for thrips control. Neonicotinoid seed treatments (such as thiamethoxam and imidacloprid), along with foliar-applied insecticides, have been widely used for thrips control in early season [16]. However, widespread and variable resistance to neonicotinoids has been reported in *Frankliniella fusca* on cotton in the Southeast and Mid-South United States [17]. Conversely, limited studies are available on the control of thrips (*F. intonsa*) during flowering and boll stages in China, except for a recent study on the application of spinosyns and diamide insecticides (e.g., spinetoram and cyantraniliprole) during flowering stage [18].

Due to variations in insecticide application history across different geographical locations, the susceptibility of thrips to insecticides varied considerably [19,20]. Recently, resistance to several insecticides (e.g., imidacloprid, flupyradifurone) has been reported in *F. intonsa* in Xinjiang cotton fields [21]. Evaluating the insecticide susceptibility of thrips will help in making informed decisions regarding appropriate chemical control strategies. Spinosyns, such as spinosad and spinetoram, are currently among the most effective insecticides for thrips control [22]. Neonicotinoids, including imidacloprid and acetamiprid, have been widely used against sucking pests on cotton [23,24,25]. We hypothesize that selection pressures in different regions lead to variations in susceptibility. In this study, we evaluated the susceptibility of *F. intonsa* populations from different geographical sites in Xinjiang to three insecticides: spinetoram, imidacloprid, and acetamiprid. The results provide a foundation for developing management strategies and aid in making decisions on appropriate chemical control of *F. intonsa* in Xinjiang.

## 2. Materials and Methods

### 2.1. Test Populations

**Figure 1:**
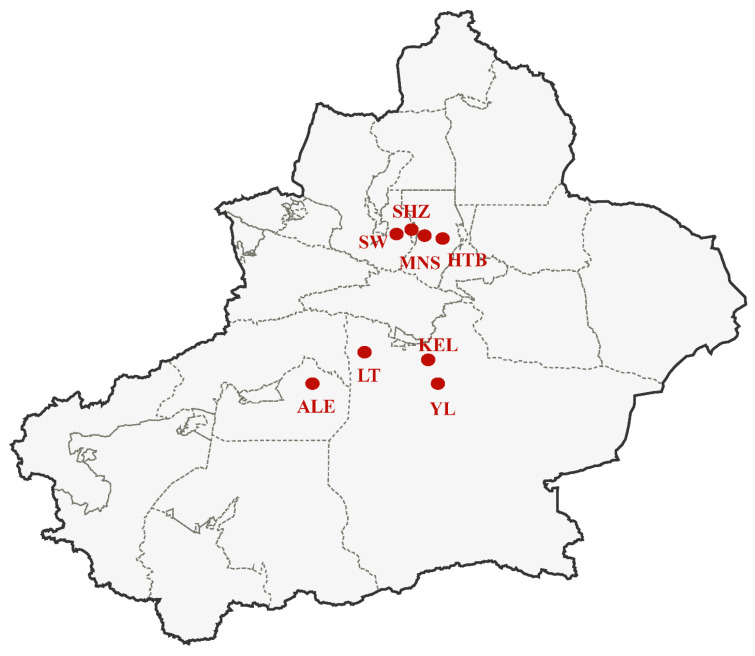
Map of sampling sites of *Frankliniella intonsa* from cotton fields in Xinjiang. ALE: Alaer, Aksu; KEL: Korla, Bayingolin; YL: Yuli, Bayingolin; LT: Luntai, Bayingolin; SW: Shawn, Tacheng; HTB: Hutubi, Changji; MNS: Manasi, Changji; SHZ: Shihezi, Shihezi.

Field *Frankliniella intonsa* populations were collected from cotton flowers at 8 geographical sites across the major cotton planting areas in Xinjiang in 2023 (Figure 1, Table 1). Cotton flowers infested with thrips were collected, temporarily maintained in glass jars in climate chambers (25 ± 1 °C, 60% ± 5% RH, 16 h: 8 h L: D photoperiod) for less than 48 h, and subsequently used for bioassays. In each location, more than 3000 thrips were collected.

### 2.2. Insecticides

Three insecticides were used in the bioassays: spinetoram 6% SC (Formulation: soluble concentrate; Trade name: Exalt; Supplier: Dow AgroSciences, Indianapolis, IN, USA; Application rate: 30~60 mg L^−1^); imidacloprid 70% WDG (Formulation: Water Dispersible Granule; Trade name: Admire; Supplier: Bayer, Leverkusen, Germany; Application rate: 35~50 mg L^−1^); acetamiprid 50% WDG (Formulation: Water Dispersible Granule; Trade name: Yicheng; Supplier: Sichuan Runer Technology, Chengdu, China; Application rate: 25~75 mg L^−1^).

### 2.3. Bioassays

A leaf-tube residue method was used to test the susceptibility of *F. intonsa* to three insecticides [26]. Each insecticide was serially diluted to five to seven concentrations with distilled water containing 0.1% Triton X-100 (Beijing Solar Bio Science and Technology Co. Ltd., Beijing, China) to obtain the mortality rate in the range of 20~80%. The concentrations of spinetoram were 0.005, 0.01, 0.05, 0.1, 0.5, 1, and 5 mg L^−1^. The concentrations of imidacloprid were 0.5, 1, 5, 10, 50, 100, and 500 mg L^−1^. The concentrations of acetamiprid were 0, 0.05, 0.1, 0.5, 1, 5, 10, and 50 mg L^−1^. A solution of distilled water with 0.1% Triton X-100 served as the control, and the control mortality rate was kept below 10%. Centrifuge tubes (1.5 mL) were treated by filling with the diluted solutions for 4 h and then dried for 2 h. The bottom of each tube was cut to create a small hole, through which thrips were aspirated into the tube by a pump. Cabbage leaf discs (*Brassica oleracea* L.) with a diameter of 1 cm were dipped into the dilutions for 10 s and dried for 30 min. The dried leaf discs were transferred individually into the tubes treated with the same concentration. Ten adult female thrips were aspirated into each tube through the hole, after which the hole was sealed with parafilm. Four to five replicates were prepared for each insecticide at each concentration. The treated thrips were placed in climate chambers (25 ± 1 °C, 60% ± 5% RH, 16 h: 8 h L: D photoperiod). Mortality was assessed 24 h after treatment. Thrips unable to move were considered dead.

### 2.4. Statistical Analysis

The mortality data were analyzed by probit analysis using POLO Plus software Version 2.0. The estimated parameters included the LC_50_ value (median lethal concentration) and LC_90_ value of active ingredient (90% lethal concentration) with 95% CI (confidence intervals) and the slope of the regression line. LC_50_ values were considered to be significantly different if their 95% CI did not overlap. Relative resistance was calculated by dividing the LC_50_ of a population by the lowest LC_50_.

## 3. Results

### 3.1. Susceptibility of Field Populations of Frankliniella intonsa to Spinetoram

**Figure 2:**
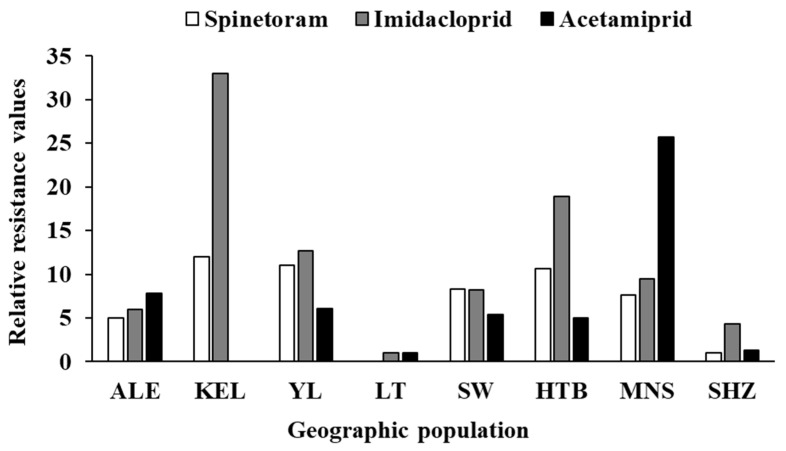
The relative resistance of different *Frankliniella intonsa* geographic populations to three insecticides. ALE: Alaer, Aksu; KEL: Korla, Bayingolin; YL: Yuli, Bayingolin; LT: Luntai, Bayingolin; SW: Shawn, Tacheng; HTB: Hutubi, Changji; MNS: Manasi, Changji; SHZ: Shihezi, Shihezi.

Field populations of *F. intonsa* from Xinjiang cotton fields showed very high susceptibility to spinetoram (Table 2). The LC_50_ values for all seven populations were below 0.05 mg L^−1^, ranging from 0.003 mg L^−1^ in Shihezi (SHZ) to 0.036 mg L^−1^ in Korla (KEL) (Table 2). The LC_90_ values ranged from 0.024 mg L^−1^ in Shihezi (SHZ) to 0.142 mg L^−1^ in Korla (KEL) (Table 2). The Shihezi (SHZ) and Alaer (ALE) populations were the most susceptible, with LC_50_ values below 0.02 mg L^−1^ and LC_90_ values below 0.1 mg L^−1^ (Table 2). Based on the overlap of 95% confidence intervals (95% CI) from the LC_50_, the SHZ population showed significantly higher susceptibility than all others. The Alaer (ALE) population was more susceptible than the KEL and Yuli (YL) populations (Table 2). Although the KEL population had the highest LC_50_ and LC_90_, there were no significant differences among KEL, YL, Shawan (SW), Hutubi (HTB) and Manasi (MNS) populations (Table 2). The greatest difference in toxicity was between populations from SHZ and KEL, with a relative resistance of 12.00 (Table 2, Figure 2).

### 3.2. Susceptibility of Field Populations of Frankliniella intonsa to Imidacloprid

Susceptibility to imidacloprid varied considerably among different field populations of *F. intonsa* in Xinjiang (Table 3). The LC_50_ values of the eight populations ranged from 4.361 mg L^−1^ in Luntai (LT) to 143.930 mg L^−1^ in Korla (KEL) (Table 3). The LC_90_ values ranged from 151.278 mg L^−1^ in Luntai (LT) to 4771.991 mg L^−1^ in Korla (KEL) (Table 3). Based on 95% CI comparisons of LC_50_, the LT population was significantly more susceptible to imidacloprid than all other populations except Shihezi (SHZ) (Table 3). The KEL population was the least susceptible, with the LC_50_ and LC_90_ values significantly higher than those of the LT, SHZ, ALE, SW and MNS populations (Table 3). No significant differences were detected in LC_50_ values among KEL, YL, and HTB populations (Table 3). The highest difference in toxicity was between the populations from LT and KEL, with a relative resistance of 33.00 (Table 3, Figure 2).

### 3.3. Susceptibility of Field Populations of Frankliniella intonsa to Acetamiprid

The susceptibility of *F. intonsa* populations to acetamiprid also varied (Table 4). The LC_50_ values of the seven populations ranged from 1.873 mg L^−1^ in Luntai (LT) to 48.154 mg L^−1^ in Manasi (MNS) (Table 4). The LC_90_ values ranged from 27.068 mg L^−1^ in Luntai (LT) to 172.306 mg L^−1^ in Manasi (MNS) (Table 4). Based on the overlap of 95% CI from the LC_50_, the LT population was significantly more susceptible to acetamiprid than all other populations except SHZ (Table 4). The least susceptible population to acetamiprid was found in the MNS population, with the LC_50_ value significantly higher than all other populations (Table 4). The highest difference in toxicity was observed between the LT and MNS populations, with a relative resistance of 25.71 (Table 4).

## 4. Discussion

As one of the most effective insecticide classes for thrips management, spinosyns have been extensively used for thrips control in China. However, resistance to spinosad and spinetoram in thrips has been widely reported in vegetable crops, especially in western flower thrips (*Frankliniella occidentalis*) [19,20,27]. In contrast, field populations of *F. intonsa* on various vegetables across China have been reported to be more susceptible to spinetoram [20,27,28]. In our study, field populations of *F. intonsa* from Xinjiang cotton fields also showed higher susceptibility to spinetoram, with LC_50_ values ranging from 0.003 mg L^−1^ to 0.036 mg L^−1^, significantly lower than the recommended dose of spinetoram (30~60 mg L^−1^). These values were lower than those reported in vegetable fields, which ranged from 0.0044 mg L^−1^ to 0.6476 mg L^−1^ [20]. The higher susceptibility observed in cotton fields compared to vegetable fields might be due to differences in insecticide selection pressure between cotton and vegetable crops. The short growth cycle and frequent cultivation of vegetable crops may lead to more frequent occurrences and intensive insecticide use, which could contribute to higher resistance levels. The high susceptibility to spinetoram observed in this study aligns with the high control efficacy (79.22% after 7 days) of spinetoram against thrips during the cotton flowering stage in Xinjiang [18]. These results suggest that spinetoram can be used for effective control of *F. intonsa* in major cotton planting areas of Xinjiang.

Imidacloprid and acetamiprid have been extensively used for controlling sucking pests (e.g., aphids and whiteflies) in cotton [24,25]. *Frankliniella intonsa* often co-occurs with cotton aphids during flowering and boll stages [29], exposing it to high insecticide selection pressure. Our results showed that the susceptibility of *F. intonsa* to imidacloprid and acetamiprid varied considerably among different field populations from Xinjiang cotton fields. The Luntai and Shihezi populations were the most susceptible to imidacloprid, while the Korla population was the least susceptible, with LC_50_ values ranging from 4.361 mg L^−1^ to 143.930 mg L^−1^. The LC_50_ values for the Korla and Manasi populations (143.930 and 41.506 mg L^−1^, respectively) reported in this study were higher than the recommended rate (35~50 mg L^−1^). Furthermore, the values also exceeded those in a previous study, in which Korla and Manasi populations had LC_50_ values of 73.58 and 12.46 mg L^−1^, respectively [21]. Similarly, Lutai and Shihezi were the most susceptible to acetamiprid, whereas the Manasi population (LC_90_ value 172.306 mg L^−1^) was the least susceptible, with its LC_90_ value higher than the recommended application rate (25~75 mg L^−1^). The susceptibility of *F. intonsa* populations in cotton fields to acetamiprid reported here (LC_50_ values from 1.873 mg L^−1^ to 48.154 mg L^−1^) was much lower than that of previously reported *F. intonsa* populations in alfalfa fields, where the LC_50_ values ranged from 1.35 mg L^−1^ to 5.52 mg L^−1^ [30], but higher than other thrips species, *F. occidentalis* and *Thrips palmi*, in vegetable fields in China [31]. These results indicate that *F. intonsa* has developed resistance to imidacloprid and acetamiprid in some cotton planting areas of Xinjiang (such as Korla and Manasi).

The variation in susceptibility of *F. intonsa* to the three insecticides among populations showed similar trends. The Lutai and/or Shihezi populations were the most susceptible populations to all three insecticides. The Korla population was the least susceptible to both spinetoram and imidacloprid, and the Manasi population was the least susceptible to acetamiprid. Similar relative resistance trends of imidacloprid and acetamiprid in the same populations (e.g., ALE, YL, SW) indicate that cross-resistance might exist in these two neonicotinoid insecticides. The reduced insecticide susceptibility in the Korla and Manasi populations emphasizes the importance of delaying the development of resistance. Judicious use of insecticides, for instance, rotating insecticides with different modes of action and applying insecticides only when necessary and at correct doses, is an effective measure for resistance management. In addition, complementing chemical control with environmentally friendly insecticides, biological control, and behavioral control would be useful strategies for long-term sustainable management of cotton thrips [32,33,34].

The resistance mechanisms of thrips to spinetoram have been linked to metabolic detoxification and target site mutations in nicotinic acetylcholine receptors [28,35]. Unlike the commonly reported target site mutation in other thrips species, no such mutations have been reported in *F. intonsa*, which aligns with its high susceptibility in the field [28]. Similarly, thrips’ resistance to neonicotinoid insecticides has also been associated with metabolic detoxification and target site mutations of nicotinic acetylcholine receptors [15,36,37]. However, the resistance mechanisms of *F. intonsa* to neonicotinoids, such as imidacloprid and acetamiprid, have been rarely studied. Investigating variations in detoxification enzyme activity and the mutation frequency at receptor target sites among different populations could provide insights into the biochemical or genetic basis of susceptibility differences in this species. It is important to note that only three insecticides were tested, and the populations were collected during a single year. Consequently, these results may not fully represent susceptibility variations over time. Ongoing monitoring of the susceptibility of *F. intonsa* populations to a wide range of insecticides across multiple years is necessary in the future.

## Figures and Tables

**Table 1:** Sample collection information for field populations of *Frankliniella intonsa* on cotton flowers.

Population	Collecting Location	Latitude (N) and Longitude (E)	Date of Collection
ALE	Alaer, Aksu	40°39′13″, 81°30′7″	August 2023
KEL	Korla, Bayingolin	41°45′0″, 85°48′36″	August 2023
YL	Yuli, Bayingolin	41°17′47″, 86°15′0″	August 2023
LT	Luntai, Bayingolin	41°59′55″, 85°6′59″	August 2023
SW	Shawan, Tacheng	44°18′47″, 85°42′6″	August 2023
HTB	Hutubi, Changji	43°49′1″, 87°33′33″	August 2023
MNS	Manasi, Changji	44°12′25″, 86°26′4″	August 2023
SHZ	Shihezi, Shihezi	44°17′15″, 85°57′38″	August 2023

**Table 2:** Susceptibility of field populations of *Frankliniella intonsa* to spinetoram.

Population	N ^a^	Slope ± SE ^b^	*χ* ^2^	*df*	LC_50_ (mg L^−1^) (95% CI ^c^)	LC_90_ (mg L^−1^) (95% CI)	Relative Resistance ^d^
ALE	340	1.991 ± 0.286	49.336	32	0.015 (0.007–0.023)	0.064 (0.043–0.111)	5.00
KEL	330	2.148 ± 0.225	43.095	31	0.036 (0.027–0.048)	0.142 (0.097–0.249)	12.00
YL	330	2.701 ± 0.403	25.241	31	0.033 (0.025–0.042)	0.099(0.074–0.155)	11.00
SW	280	4.078 ± 0.718	28.257	26	0.025 (0.018–0.030)	0.051(0.042–0.070)	8.33
HTB	280	4.190 ± 0.811	22.981	22	0.032 (0.021–0.040)	0.065 (0.053–0.086)	10.67
MNS	350	3.413 ± 0.320	65.172	33	0.023 (0.019–0.028)	0.056 (0.045–0.075)	7.67
SHZ	280	1.471 ± 0.356	38.920	26	0.003 (0.000–0.006)	0.024 (0.013–0.056)	1

^a^ Number of thrips tested. ^b^ Standard error. ^c^ 95% confidence intervals. ^d^ LC_50_ of a population/the lowest LC_50_.

**Table 3:** Susceptibility of field populations of *Frankliniella intonsa* to imidacloprid.

Population	N ^a^	Slope ± SE ^b^	*χ* ^2^	*df*	LC_50_ (mg L^−1^) (95% CI ^c^)	LC_90_ (mg L^−1^) (95% CI)	Relative Resistance ^d^
ALE	340	1.789 ± 0.190	61.855	32	26.178 (16.795–39.586)	136.182 (82.272–303.500)	6.00
KEL	220	0.843 ± 0.145	43.765	20	143.930 (72.428–499.435)	4771.991 (835.222–58,955.337)	33.00
YL	350	0.948 ± 0.109	47.730	33	55.240 (31.718–103.365)	1242.643 (495.244–5713.300)	12.67
LT	350	0.832 ± 0.098	49.699	33	4.361(2.080–7.895)	151.278 (66.495–579.959)	1
SW	270	1.274 ± 0.181	15.804	25	35.701 922.918–54.178)	360.664 (198.691–1039.698)	8.19
HTB	250	1.832 ± 0.507	24.700	23	82.261 (30.081–130.056)	412.004 (235.305–2393.660)	18.86
MNS	340	1.463 ± 0.202	21.871	32	41.506 (28.714–58.674)	312.125 (188.553–694.108)	9.52
SHZ	270	0.692 ± 0.117	24.700	23	18.872 (5.953–53.825)	1342.636 (275.169–106,949.366)	4.33

^a^ Number of thrips tested. ^b^ Standard error. ^c^ 95% confidence intervals. ^d^ LC_50_ of a population/the lowest LC_50_.

**Table 4:** Susceptibility of field populations of *Frankliniella intonsa* to acetamiprid.

Population	N ^a^	Slope ± SE ^b^	*χ* ^2^	*df*	LC_50_ (mg L^−1^) (95% CI ^c^)	LC_90_ (mg L^−1^) (95% CI)	Relative Resistance ^d^
ALE	340	3.384 ± 0.629	70.220	32	14.613 (9.031–24.157)	34.948 (21.812–127.163)	7.80
YL	350	1.989 ± 0.219	25.300	33	11.394 (8.549–14.949)	50.214 (35.635–80.462)	6.08
LT	350	1.105 ± 0.131	25.413	33	1.873 (1.139–2.802)	27.068 (16.421–55.106)	1
SW	180	2.139 ± 0.381	46.202	16	10.013 (4.213–20.005)	39.778 (19.934–457.314)	5.35
HTB	280	1.637 ± 0.179	86.891	26	9.347 (4.352–17.735)	56.714 (28.016–211.050)	4.99
MNS	270	2.315 ± 0.407	26.988	25	48.154 (31.483–66.223)	172.306 (117.382–346.374)	25.71
SHZ	270	0.961 ± 0.142	52.606	25	2.403 (0.446–5.952)	51.743 (221.227–256.909)	1.283

^a^ Number of thrips tested. ^b^ Standard error. ^c^ 95% confidence intervals. ^d^ LC_50_ of a population/the lowest LC_50_.

## Data Availability

The data presented in this study are fully contained within the figures of the article. Further inquiries can be directed to the corresponding authors.
